# *Tithonia diversifolia* as a Supplementary Feed for Dairy Cows

**DOI:** 10.1371/journal.pone.0165751

**Published:** 2016-12-01

**Authors:** Rafael Sandin Ribeiro, Stephanie Amelia Terry, João Paulo Sacramento, Sylvia Rocha e Silveira, Cláudia Braga Pereira Bento, Elsa Fernandes da Silva, Hilário Cuquetto Mantovani, Marco Antônio Sundfeld da Gama, Luiz Gustavo Ribeiro Pereira, Thierry Ribeiro Tomich, Rogério Martins Maurício, Alexandre Vieira Chaves

**Affiliations:** 1 Bioengineering Department, Universidade Federal de São João del-Rei, São João del-Rei, MG, Brazil; 2 The University of Sydney, Faculty of Veterinary Science, School of Life and Environmental Sciences, Sydney, NSW, Australia; 3 Departamento de Microbiologia, Universidade Federal de Viçosa, Viçosa, MG, Brazil; 4 Embrapa Gado de Leite, Juiz de Fora, MG, Brazil; Leibniz-Institut fur Pflanzengenetik und Kulturpflanzenforschung Gatersleben, GERMANY

## Abstract

The objective of this study was to examine the effects of *Tithonia diversifolia* as a supplementary forage on dairy cow performance and methane production. Nine lactating Holstein × Zebu dairy cows (519 ± 53.3 kg of body weight and 66 ± 13.3 d in milk) were paired by milk yield (21.3 ± 2.34 kg/d) and body weight and randomly assigned to three dietary treatments in a Latin square design with 21-d experimental periods (14 d for diet adaptation and 7 d for measurements and sample collection). The dietary treatments included the control diet consisting of fresh sugar cane plus concentrate (44:56, % of diet DM), and two treatment diets containing different levels of fresh *T*. *diversifolia* (6.5 and 15.4%, DM basis) which partially replaced both sugarcane and concentrates. Methane production was measured using the sulphur hexafluoride (SF_6_) technique from d 16 to d 21 of each experimental period. Analysis of the gas samples was performed by gas chromatography. The inclusion of *T*. *diversifolia* at 15.4% DM had no effects on DM intake, milk production, nitrogen balance or methane production. There was no effect on the concentrations of total saturated fatty acids (SFA), monounsaturated fatty acids (MUFA), and polyunsaturated fatty acids (PUFA) in milk fat (*P* ≥ 0.28), though individual milk fatty acids were affected. Serum concentrations of glucose, urea nitrogen (BUN), triglycerides, β-hydroxybutyrate (BHBA), and cholesterol were unaffected by the dietary treatments (*P* ≥ 0.13). There was a time (2 and 6 h post-feeding) and dietary treatment effect (*P* < 0.01) on the acetate to propionate ratio in the rumen. A denaturing gradient gel electrophoresis analysis of the archaeal community showed distinct clustering of the archaea populations for control and treatment diets. Taken together, our results indicate the potential of *T*. *diversifolia* as a supplementary forage for dairy cattle in the tropics.

## Introduction

The inclusion of concentrated feeds into a dairy cattle diet is an expensive necessity, which according to recent estimates from Embrapa Dairy Research can correspond up to approximately 40% of milk production costs across dairy farming systems (http://www.cileite.com.br/). Whilst it is well established that an increase of concentrates in feed, leads to increases in animal production, this may be impractical for farms in the tropics due to the high cost of concentrates. Therefore it is more viable to investigate and recommend affordable feed options that mimic the effects of concentrates without any adverse effects on animal productivity and enteric methane production.

*Tithonia diversifolia*, a tropical shrub native to Central America, presents some unique characteristics that could be of interest in ruminant production systems. Compared to other tropical forages commonly used for milk production, *T*. *diversifolia* has greater protein and phosphorus content [1–3]. Additionally, some studies have shown that the nutritive value of *T*. *diversifolia* is kept relatively constant throughout the dry season [4,5], while the nutritional quality of most tropical grasses markedly decreases under water limited conditions [2]. Additionally, *T*. *diversifolia* is a robust weed which is easily grown and can aid in soil rejuvenation [6]. Furthermore, *T*. *diversifolia* has previously showed potential as a methane reducing feed additive in ruminants [7] thus, it has been suggested that *T*. *diversifolia* could be used as an alternative forage source in tropical regions.

The importance of *T*. *diversifolia* as a potential methane reducer is in the fact that agricultural methane emissions are responsible for approximately 10–12% of global anthropogenic emissions [8,9]. Methane has 25 times the global warming potential of carbon dioxide (CO_2_), and its production by ruminants is estimated to represent an energy loss between 2–12% of total energy intake [10]. With increasing pressure from the global community to reduce methane emissions and the inverse correlation between energy utilization and CH_4_ production, the supplementation of ruminant diets with *T*. *diversifolia* has been suggested as a promising dietary strategy. Currently, *T*. *diversifolia* has been reported to reduce methane output by 6-fold when compared to a control due to the presence of secondary metabolites in the plant [7]. Anti-nutrients such as tannins and saponins have been shown to decrease methane production due to their inhibitory effects on rumen ciliate protozoa [7,11].

Comparably, some studies have shown that these secondary compounds may also result in disadvantageous effects on animal performance, which may limit the use of *T*. *diversifolia* as an alternative feed in ruminant production systems [12]. However, Mahecha et al. [13] found that *T*. *diversifolia* replaced up to 35% dry matter (DM) of concentrates in the diet of dairy cows without any adverse effects on milk production and composition. In addition, a recent *in vitro* study by Terry et al. [14] indicated that replacing up to 15.2% of sugarcane and up to 14% of concentrates (DM basis) with *T*. *diversifolia* increased (P < 0.01) the production of total volatile fatty acids (VFA), which may indicate improved animal performance in field conditions. Possible dietary effects on milk fatty acid composition are also of interest as a number of *in vitro* and animal studies have shown that ruminant milk fat contains a number of bioactive fatty acids with potential health-promoting effects [15], which is supported by a recent review of observational studies on the relationship between dairy fat consumption, obesity, and cardio metabolic disease [16].

The aim of this study was to examine the effects of dietary *T*. *diversifolia* on performance, milk composition, and methane production with a specific investigation into rumen fermentation (e.g: VFA and ammonia), nitrogen (N) balance and archaeal diversity of dairy cows fed sugarcane-based diets. It was hypothesized that the inclusion of *T*. *diversifolia* would have no effect on milk production, milk composition, N balance nor methane production, however *T*. *diversifolia* would affect rumen fermentation and archaea diversity.

## Materials and Methods

These experiments were conducted at the Universidade Federal de São João Del-Rei (UFSJ), São João Del-Rei, Minas Gerais, Brazil (21° 05' 11.20" S, 044° 13' 35.30" W). The cows were cared for in accordance with the guidelines of the Universidade Federal de São João del-Rei Animal Care and Use Committee (Approved protocol number 14/2014).

### Experimental design and treatments

Nine lactating Holstein × Zebu dairy cows (519 ± 53.3 kg of BW and 66 ± 13.3 DIM) were paired by milk yield (21.3 ± 2.34 kg/d) and body weight and randomly assigned to three dietary treatments in a 3 × 3 Latin square design with three, 21 d-experimental periods. The first 14 d of each period were used for diet adaptation and the last 7 d for measurements and sample collection. Two non-lactating, ruminally cannulated heifers were randomly assigned by period to individual dietary treatments to provide ruminal fluid for volatile fatty acid (VFA), ammonia and denaturing gradient gel electrophoresis analyses (DGGE).

The treatments included the control diet consisting of fresh sugar cane supplemented with a concentrate mixture and 2 treatment diets containing 6.5 and 15.4% (DM basis) of *T*. *diversifolia* which replaced up to 20.8% of sugarcane and up to 11.4% of concentrates (Table 1). The diets were fed as total mixed rations (TMR) and formulated using the Large Ruminant Nutrition System (version 1.0.29) to meet the nutrient and energy requirements for maintenance and milk production (20 kg/day).

**Table 1:** Ingredients and chemical composition of the experimental diets and *Tithonia diversifolia*.

			Diets^1^		
Item		*Tithonia diversifolia*	Control	6.5%T	15.4%T
Ingredients, % diet DM					
	Fresh *T*. *diversifolia*		0	6.5	15.4
	Fresh Sugarcane		43.8	40.1	34.7
	Ground corn		31.2	30.2	29.1
	Soybean meal		24.2	22.4	20
	Limestone		0.79	0.79	0.79
Dietary chemical composition					
	Dry matter, %		63.8	61.6	59.0
	Crude protein, % DM		18.5	18.4	18.4
	Neutral detergent fiber, % DM		29.4	30.6	32.2
	Acid detergent fiber, % DM		15.1	16.4	18.2
	Ether extract, % DM		1.4	1.4	1.4
	NFC^2^		46.1	44.8	42.9
	Ash, % DM		4.5	4.8	5.1
Orts chemical composition					
Dry matter, %			29.3	29.2	29.4
Crude protein, % DM			14.5	14.4	13.8
Neutral detergent fiber, % DM			39.5	39.8	43.5
Acid detergent fiber, % DM			20.8	22.3	26.2
Ash, % DM			5.0	5.2	5.4
Fatty acid composition (g/100 g)				
	C12:0 Lauric acid	0.22			
	C14:0 Myristic acid	0.84			
	C16:0 Palmitic acid	31.0			
	C18:0 Stearyl acid	3.32			
	C18:1n-9 Oleic acid	4.47			
	C18:2n-6 Linoleic acid	23.9			
	C18:3n-3 Linoleic acid	15.8			
	C20:0 Arachonic acid	2.77			
	C22:0 Behenic acid	3.26			
	C24:0 Lignoceric acid	1.78			
	Total Relative (%)	87.4			

^1^Control: 0% *Tithonia diversifolia*, 6.5%T: 6.5% *Tithonia diversifolia*, 15.4%T: 15.4% *Tithonia diversifolia*

^2^NFC = non-fibrous carbohydrates [NFC = 100 − (CP + NDF + EE + ash)]

### Feed intake

Cows were kept in individual tie stalls and offered the diets twice daily at 0900 h and 1600 h (5 to 10% orts). The forage ingredients were prepared fresh daily, being cut and then chopped to 1.5 cm in length. Concentrates (corn, soybean meal and minerals) were mixed using a feed mixer with 300 kg of capacity (Model MIN 300C, Incomagri Ltd, Itapira-SP, Brazil) before being offered to the animals. The forages and concentrates were mixed into individual large bags prior to feeding. The animals were then fed the treatments as a TMR respective of the given experimental diet of each cow. Feed offered and refused was recorded each day and adjusted during the transition period so that there was a 10% excess of feed. Samples of feed and orts were dried at 135°C for 2 h every day for the determination of dry matter content.

### Milk yield and composition

Cows were milked twice daily at 0700 and 1400 h, with both a.m. and p.m. sampling occurring on d 17 to d 19 in each period for the determination of milk yield and composition. Individual a.m. and p.m. milk samples were used for the determination of milk composition. A bronopol tablet (D & F Control Systems Inc., San Ramon, CA) was used as a preservative, with milk being kept at 4°C until analysis at EMBRAPA-CNPGL (Dairy Research Centre, Juiz de Fora, MG, Brazil). Milk was analyzed for fat, lactose and protein with an infrared analyzer (Bentley2000, Bently Instruments). Milk urea nitrogen (MUN) concentration was analyzed by the calorimetric method using a commercial kit (Sigma Diagnostics, St. Louis, MO).

Milk fatty acid composition was determined in both a.m. and p.m. milkings from samples collected on d 17 of each period. Milk samples were thawed at room temperature and a volume of 1 mL was used for lipid extraction using a mixture of diethyl ether and hexane according to AOAC [17] (Method 989.05). The organic phase containing the milk fat (~20 mg) was evaporated to dryness at 40°C under oxygen-free nitrogen. Fatty acid methyl esters (FAME) were obtained by base-catalyzed transmethylation as described in detail by Baldin et al. [18]. Fatty acid methyl esters were quantified by a gas chromatograph (model 7820-A, Agilent Technologies) fitted with a flame-ionization detector and equipped with a CP-Sil 88 fused-silica capillary column (100 m × 0.25 mm × 0.2 μm film thickness; Varian Inc). Operating conditions were set as described by Cruz-Hernandez et al. [19]. The FAME were identified by comparison of retention times with commercial FAME standards; minor trans/cis-C18:1 isomers and trans-9 cis-11 CLA were identified according to their order of elution reported under the same GC conditions [18]. Milk fatty acid composition was expressed as a weight percentage of total fatty acids using theoretical relative response factors [20].

### Blood metabolites

Blood was taken from the mammary vein of all cows on day 19 at 2 and 6 h after daily feed delivery. Samples were collected using 4 mL vacutainer tubes (13×75 mm; BD vacutainer–Plus blood collection tubes Part No. 368521 –Becton Dickinson, Belliver Industrial Estate, Plymouth, UK). Samples were processed at the laboratory where they were centrifuged (1800 × g, 20 min, +4°C) and plasma was harvested and stored at -2° C for later analysis of glucose, BUN, triglycerides, cholesterol, BHBA and NEFA.

### N balance

During the measurement period, total collections of feces and urine were taken over 3 consecutive days (d 15 to 17) for the quantification of N content as determined by the Kjeldahl method. Urine was collected once a day for the quantification of total N content (AOAC, [21]; method 954.01) with a part of the sample being acidified with H_2_SO_4_ for creatinine concentration determination as described by Broderick and Radloff [22]. Both samples were frozen and stored at -20°C until analysis. Daily urine volume (DUV, kg/day) was estimated from BW and urinary creatinine concentration [23] as:

$$DUV=BW\times \frac{29}{Urinary\;concentration\;of\;creatinine\;(mg/L)}$$

Fecal samples were collected twice daily at 0900 and 1600 h. Samples were collected from the rectal ampulla and immediately frozen at -20°C. For analysis, fecal samples were dried at 55°C for 72 h and ground to 1 mm. For quantification of total nitrogen fecal samples corresponding to collection of morning and afternoon were combined. Total fecal output was estimated by acid-insoluble ash analysis [24].

### Rumen Fluid

Rumen contents (~200 mL total) were collected from each cow via the ruminal fistula 2 and 6 h after morning feeding. The fluid was pooled and filtered through 4 layers of cheesecloth and pH was measured. The ruminal fluid was immediately transported, within 5 minutes from collection, to the laboratory. Rumen liquor was transferred into 50 mL centrifuge tubes. The tubes were centrifuged at 500 × *g* for 5 minutes at 5°C (Novatecnica company, model NT 825, Brazil) and then centrifuged at 4,500 × *g* for 15 minutes at 5°C for the determination of VFA. The samples were then acidified and spun again at 12,000 × *g* for 10 min and the cell-free supernatants were treated as described by Siegfried et al. [25].

Volatile fatty acids were determined by HPLC in a Dionex Ultimate 3000 Dual detector HPLC (Dionex Corporation, Sunnyvale, CA, USA) coupled to a refractive index (RI) Shodex RI-101 maintained at 40°C using a ion exchange column Phenomenex Rezex ROA, 300 × 7.8 mm maintained at 45°C. Mobile phase was prepared with 5 mmol/L of sulfuric acid (H_2_SO_4_) and the flow was set to 0.7 mL/min.

The following VFA were used for the calibration of the standard curve: acetic, succinic, formic, propionic, valeric, isovaleric, isobutyric and butyric acid. All acids were prepared with a final concentration of 10 mmol/L, except isovaleric acid (5 mmol/L) and acetic acid (20 mmol/L).

Ammonia was determined by the colorimetric method of Chaney and Marbach [26]. Absorbance was measured at 630 nm in a Spectronic 20D spectrophotometer (Thermo Fisher Scientific, Madison, WI, USA) and ammonium chloride (NH_4_Cl) was used as standard. Total ammonia was expressed in mmol/L at times 2 hours and 6 hours.

### Methane Measurement

Methane gas production was measured using the sulphur hexafluoride (SF6) tracer gas method as modified and described by Berndt et al. [27]. On d 14 of the first period of evaluation, the brass permeation tubes containing SF6 with a pre-determined release rate of 2.63 ± 0.94 mg/d (average ± SD) were placed in the rumen of each cow. Samples (7.5 mL) of respired air over a 24 h period were taken up by an evacuated PVC yolk (Labco Ltd., Buckinghamshire, UK) over six consecutive days (d 16 to d 21) in each period. After samples of gas had been collected, N_2_ was injected into the PVC container so that the internal pressure became positive, allowing withdrawal of the gas. Analysis of the gas samples was performed by gas chromatography.

### Denaturing Gradient Gel Electrophoresis

Rumen fluid (50 mL) was collected from the 2 ruminally cannulated heifers at each collection period at each treatment diet for a denaturing gel gradient electrophoresis. Samples were pooled for each treatment and stored at -80°C. For DNA extraction, samples were thawed at room temperature and processed as described by Stevenson and Weimer [28]. Polymerase chain reactions were performed using a Biocycler MG96G with the archaeal primers Arch21f (5′-TTCCGGTTGATCCYGCCGGA-3′) and Arch958r (5′-YCCGGCGTTGAMTCCAATT-3′) as described by Muhling et al. [29].

### Chemical analysis

Diets and orts were analyzed for DM (method 967.03), ash (method 942), ADF (method 973.18), and ether extract contents following AOAC [21] methods. Ether extract (EE) content was only determined in the diets ingredients by extraction with petroleum ether using a Soxtherm Fat Extractor (Gerhardt Instruments, Germany; method 920.39). Neutral detergent fiber (NDF) content was analyzed according to Van Soest et al. [30] without the use of sodium sulphite or heat stable α-amylase. N concentration was determined by the Kjeldahl method (Vapodest 20S, Gerhardt Instruments, Germany) with CP content calculated as *N* × 6.25. Non-fibrous carbohydrates (NFC, % of DM) was calculated using the equation:

NFC=100−(CP+NDF+EE+Ash)

The fatty acid composition of *Tithonia diversifolia* was determined using the one-step procedure originally described by Sukhija and Palmquist [31] with adaptations [32]. The FAME were analyzed using a gas chromatograph (model 6890 N, Agilent Technologies) equipped with a flame-ionization detector and fitted with a highly polar capillary column (HP-FFAP, 25m × 0.2mm × 0.33μm, Agilent Technologies). Operating conditions were described in detail elsewhere [33]. Peaks were identified by retention time comparisons with a commercial standard containing a mixture of FAME (Supelco 37, #47885-U).

### Statistical analysis

Data (feed intake, milk yield and composition, blood, and CH_4_) were analyzed as a 3 × 3 Latin square design using the mixed model procedure of SAS (SAS Inc., 2016; SAS OnlineDoc 9.1.3. Cary, NC, USA). Degrees of freedom were adjusted using the Kenward-Roger option. Means were compared using the LSMEANS/DIFF command with dietary treatment and period as fixed terms and cow nested within a period and within a sequence as a random effect. Sampling day was analyzed as a repeated measure. Covariance structures were specified to allow for repeated measures over days. When there was a difference among treatments (*P* < 0.05), orthogonal polynomial contrasts were performed to test for linear and quadratic responses to increasing amounts of *T*. *diversifolia* replacing feed ingredients in the diet. Ruminal VFA data were analyzed with a similar model but means were compared using the LSMEANS/DIFF command with dietary treatment as fixed terms and fistulated cow as a random effect. For the DGGE analysis Dice’s similarity coefficient (D_sc_) was used to compare the data sets with an optimization of 1% and a tolerance of 1% [34]. Clustering was performed using the unweighted pair group method (UPGMA).

## Results

The nutrient composition of the ingredients and their proportions in the diet are reported in Table 1. Feed intake did not differ (*P =* 0.96) among dietary treatments (Table 2). Supplementation with *T*. *diversifolia* had no effect (*P* ≥ 0.74) on milk yield or milk component yields and concentrations of fat, protein, lactose, or urea.

**Table 2:** Dry matter intake, milk yield and milk composition of dairy cows fed two concentrations of *T*. *diversifolia* in sugarcane-based diets.

	TREATMENTS^1^				
	Control	6.5%T	15.4%T	SEM	*P*-value
DMI, kg/d	18.6	18.9	18.7	0.63	0.96
Milk yield, kg/d	22.7	23.1	22.8	1.49	0.98
Milk fat, %	3.58	3.48	3.45	0.127	0.76
Milk protein, %	3.23	3.15	3.08	0.082	0.48
Milk lactose, %	4.34	4.42	4.36	0.106	0.84
Milk urea, %	13.8	13.5	12.9	0.83	0.74

^1^Control: 0% *Tithonia diversifolia*, 6.5%T: 6.5% *Tithonia diversifolia*, 15.4%T: 15.4% *Tithonia diversifolia*

Milk fatty acid composition is presented in Table 3. The inclusion of *T*. *diversifolia* had no effect (*P* ≥ 0.28) on the milk fat concentrations of total SFA, MUFA, or PUFA for either a.m. or p.m. milking. However, there was a general trend towards lower concentrations of linear odd-chained, and increased contents of iso-branched fatty acids, *trans*-C18:1, and non-conjugated C18:2 isomers in milk fat as *T*. *diversifolia* concentrations increased in the diet. Consistent with the increment in *trans*-11 C18:1 content, the concentration of *cis*-9, *trans*-11 CLA was also increased in milk fat from cows fed *T*. *diversifolia*.

**Table 3:** Milk fatty acid composition (g/100g of total FA) of both a.m. and p.m. milkings from dairy cows fed two concentrations of *T*. *diversifolia* in sugarcane-based diets.

	AM Milking					PM Milking				
	Treatment^1^			SEM	P-value	Treatment			SEM	P-value
Fatty Acid (FA)	Control	6.5% T	15.4% T			Control	6.5% T	15.4% T		
C4:0	3.73	3.70	3.81	0.11	0.76	3.59	3.56	3.54	0.11	0.96
C5:0	0.05	0.04	0.04	0.004	0.07	0.05^a^	0.04^a^^b^	0.03^b^	0.004	0.02
C6:0	2.62	2.62	2.61	0.07	1.00	2.51	2.52	2.45	0.07	0.73
C7:0	0.06	0.05	0.04	0.01	0.16	0.06	0.05	0.04	0.005	0.06
C8:0	1.72	1.75	1.66	0.06	0.58	1.64	1.68	1.60	0.07	0.68
C9:0	0.09	0.08	0.06	0.01	0.16	0.08	0.08	0.06	0.01	0.06
C10:0	4.27	4.35	3.98	0.20	0.41	4.06	4.17	3.79	0.20	0.40
C10:1 *c*-9	0.42	0.39	0.38	0.02	0.41	0.44	0.41	0.40	0.02	0.35
C11:0	0.16	0.15	0.12	0.02	0.14	0.16	0.15	0.11	0.01	0.10
C12:0	5.00	5.04	4.56	0.25	0.33	4.89	4.97	4.48	0.26	0.37
C14:0	12.67	12.71	12.55	0.46	0.97	12.48	12.72	12.48	0.49	0.93
C14:0 *iso*	0.08^c^	0.09^b^	0.12^a^	0.003	< .0001	0.08^b^	0.09^b^	0.13^a^	0.004	< .0001
C14:1 *c*-9	1.12	1.04	1.07	0.07	0.72	1.24	1.14	1.19	0.07	0.618
C15:0	1.65	1.41	1.35	0.11	0.13	1.71	1.44	1.34	0.11	0.08
C15:0 *anteiso*	0.44	0.44	0.45	0.01	0.61	0.44	0.44	0.47	0.02	0.42
C15:0 *iso*	0.22^b^	0.21^b^	0.25^a^	0.01	0.02	0.21^b^	0.21^b^	0.25^a^	0.01	0.004
C16:0	30.0	28.9	29.4	0.59	0.48	30.3	29.1	28.9	0.76	0.36
C16:0 iso	0.17^b^	0.18^b^	0.21^a^	0.01	0.0002	0.17^b^	0.17^b^	0.21^a^	0.01	0.001
C16:1 *t*-12	0.18	0.17	0.18	0.02	0.91	0.18	0.19	0.18	0.01	0.79
C16:1 *c*-9 + C17:0 *anteiso*	1.41	1.35	1.36	0.07	0.80	1.55	1.44	1.46	0.07	0.52
C16:1 *t*-9 + C17:0 *iso*	0.36	0.36	0.37	0.02	0.98	0.36	0.36	0.37	0.02	0.92
C17:0	0.52	0.45	0.48	0.03	0.29	0.56	0.51	0.45	0.03	0.08
C17:1 *c-9*	0.17	0.16	0.16	0.01	0.72	0.20	0.18	0.18	0.01	0.48
C18:0	8.28	8.79	8.98	0.46	0.55	7.99	8.58	8.91	0.47	0.39
C18:0 *iso*	0.05	0.05	0.05	0.00	0.79	0.05	0.05	0.05	0.01	0.63
C18:1 *c*-9	15.2	15.4	15.6	0.68	0.92	15.6	16.0	16.7	0.86	0.67
C18:1 *c*-11	0.43	0.45	0.48	0.03	0.46	0.46	0.47	0.51	0.03	0.48
C18:1 *c*-12	0.16	0.16	0.16	0.01	0.87	0.16	0.16	0.17	0.01	0.70
C18:1 *c*-13	0.06^b^	0.07^a^^b^	0.08^a^	0.003	0.04	0.061^b^	0.065^a^^b^	0.073^a^	0.003	0.03
C18:1 *t*-4	0.019^b^	0.021^a^^b^	0.024^a^	0.001	0.01	0.018^b^	0.021^a^^b^	0.023^a^	0.001	0.02
C18:1 *t*-5	0.018	0.018	0.021	0.001	0.11	0.02	0.02	0.02	0.001	0.20
C18:1 *t*-6,*t*-8	0.20^b^	0.23^b^	0.28^a^	0.01	0.005	0.19^b^	0.22^a^^b^	0.25^a^	0.02	0.04
C18:1 *t*-9	0.15^c^	0.18^b^	0.21^a^	0.01	0.001	0.14^b^	0.18^a^	0.20^a^	0.01	0.0004
C18:1 *t*-10	0.28	0.32	0.34	0.02	0.19	0.32	0.30	0.33	0.04	0.80
C18:1 *t*-11	0.74^b^	0.87^b^	1.04^a^	0.06	0.004	0.71^b^	0.82^b^	0.98 ^a^	0.05	0.002
C18:1 *t*-12	0.18^c^	0.22^b^	0.28^a^	0.02	0.001	0.17^b^	0.22^a^	0.26^a^	0.02	0.001
C18:1 *t*-13,*t-*14	0.24^b^	0.28^b^	0.36^a^	0.02	0.001	0.19^b^	0.20^b^	0.27^a^	0.02	0.004
C18:1 *t*-16	0.16^c^	0.23^b^	0.27^a^	0.01	< .0001	0.16^c^	0.21^b^	0.26^a^	0.01	0.0003
C20:1 *c*-11	0.04	0.04	0.04	0.003	0.53	0.04	0.04	0.04	0.003	0.35
C18:2 *c*-9,*t*-12	0.025^b^	0.029^a^^b^	0.032^a^	0.001	0.002	0.024^b^	0.028^b^	0.032^a^	0.001	0.002
C18:2 *n*-6	2.65	2.69	2.33	0.14	0.16	2.69	2.77	2.46	0.16	0.36
C18:2 *t*-9, *c*-12	0.02^b^	0.03^a^	0.03^a^	0.001	0.0002	0.02^b^	0.02^b^	0.03^a^	0.001	0.001
C18:2 *t*-9, *t*-12	0.01	0.02	0.02	0.001	0.14	0.01^b^	0.01^b^	0.02^a^	0.001	0.02
C18:3 *n*-3	0.23	0.23	0.25	0.02	0.55	0.22	0.25	0.26	0.02	0.31
C18:3 *n*-6	0.04	0.04	0.03	0.002	0.30	0.04	0.04	0.03	0.003	0.22
C19:0 + C18:1 *c*-15	0.08^b^	0.09^b^	0.10^a^	0.003	0.001	0.08^b^	0.09^b^	0.11^a^	0.004	0.0001
C20:2 *n*-6	0.03	0.03	0.03	0.001	0.46	0.03	0.03	0.03	0.002	0.92
C20:3 *n*-6	0.10	0.11	0.10	0.01	0.72	0.11	0.10	0.10	0.01	0.97
C20:4 *n*-6	0.19	0.19	0.17	0.01	0.49	0.20	0.20	0.19	0.01	0.58
C20:5 *n*-3	0.02	0.02	0.02	0.001	0.56	0.02	0.02	0.02	0.001	0.36
C22:5 *n*-3	0.05	0.05	0.05	0.004	0.99	0.05	0.05	0.05	0.005	0.98
CLA *c*-9, *t*-11	0.40^b^	0.42^a^^b^	0.50^a^	0.03	0.04	0.41^b^	0.43^b^	0.53^a^	0.03	0.02
CLA *t*-9, *c*-11	0.01	0.01	0.01	0.001	0.94	0.010^b^	0.012^a^^b^	0.015^a^	0.001	0.01
CLA *t*-10, *c*-12	0.01	0.01	0.01	0.001	0.99	0.005^b^	0.005^b^	0.007^a^	0.0004	0.002
C12:1 *c*-9 + C13:0	0.39	0.34	0.30	0.03	0.08	0.40	0.35	0.31	0.03	0.07
Total SFA	72.0	71.3	71.0	0.93	0.73	71.3	70.7	69.5	1.14	0.54
Total MUFA	20.2	20.7	21.5	0.79	0.52	20.8	21.3	22.6	0.96	0.39
Total PUFA	3.36	3.42	3.06	0.16	0.28	3.42	3.54	3.22	0.19	0.48

^a,b,c^Means within a row followed by different superscript letters differ significantly (*P* < 0.05).

^1^Control: 0% Tithonia diversifolia, 6.5%T: 6.5% Tithonia diversifolia, 15.4%T: 15.4% Tithonia diversifolia

**Fig 1:**
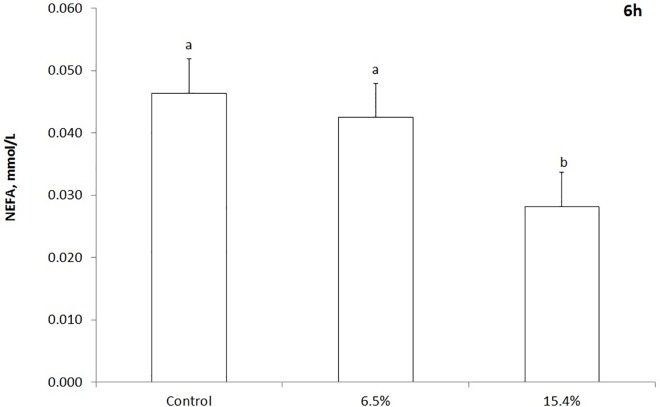
Concentration of NEFA in blood of dairy cows fed different concentrations of *T*. *diversifolia* 6 h after feeding.

There was no treatment effect (*P > 0*.*5*) of *T*. *diversifolia* at either of the inclusion rates on serum glucose, BUN, triglycerides or cholesterol concentrations (Table 4). The average concentrations of serum glucose, BUN, triglycerides and cholesterol were 40.6, 28.4, 10.4 and 103.8 mg/dL, respectively. The inclusion of *T*. *diversifolia* also had no effect (*P* = 0.64) on the concentration of BHBA. There was a treatment × time interaction effect (*P* < 0.001) for the concentration of NEFA (Fig 1). At 6 h, the concentration of NEFA was lower at a 15.4% *T*. *diversifolia* inclusion rate than the other treatments (Fig 1). There was no effect (*P* ≥ 0.51) of *T*. *diversifolia* on N intake, excretion or balance (Table 5).

**Table 4:** Concentrations of blood metabolites in dairy cows fed two concentrations of *T*. *diversifolia* in sugarcane-based diets.

	TREATMENTS^1^				*P*-value		
Item^2^	Control	6.5%T	15.4%T	SEM	Treat	Time	Treat × Time
Glucose, mg/dL	39.8	41.5	40.4	1.03	0.50	<0.0001	0.14
BUN, mg/dL	27.9	30.2	27.1	1.89	0.50	<0.0001	0.13
Triglycerides, mg/dL	9.9	10.1	11.2	0.7	0.39	0.33	0.43
Cholesterol, mg/dL	95.2	102.9	113.2	9.06	0.39	0.55	0.20
BHBA, mmol/L	0.89	0.68	0.81	0.161	0.64	0.74	0.89
NEFA, mmol/L	0.034	0.032	0.028	0.004	0.47	<0.0001	0.001

^1^Control: 0% *Tithonia diversifolia*, 6.5%T: 6.5% *Tithonia diversifolia*, 15.4%T: 15.4% *Tithonia diversifolia*

^2^BHBA = beta-hydroxybutyrate; NEFA = non esterified fatty acids.

**Table 5:** Nitrogen (N) intake and retention in dairy cows fed two concentrations of *T*. *diversifolia* in sugarcane-based diets.

	TREATMENTS^1^				
Item	Control	6.5% T	15.4% T	SEM	*P-*value
N intake (g/day)	563.1	564.1	557.2	28.0	0.9822
Fecal N (g/day)	194.0	201.1	197.8	13.0	0.9293
Urinary N (g/day)	61.8	56.3	63.7	4.64	0.5134
Total N excreted (g/day)	255.9	257.4	261.5	14.2	0.9592

^1^Control: 0% *Tithonia diversifolia*, 6.5%T: 6.5% *Tithonia diversifolia*, 15.4%T: 15.4% *Tithonia diversifolia*

**Fig 2:**
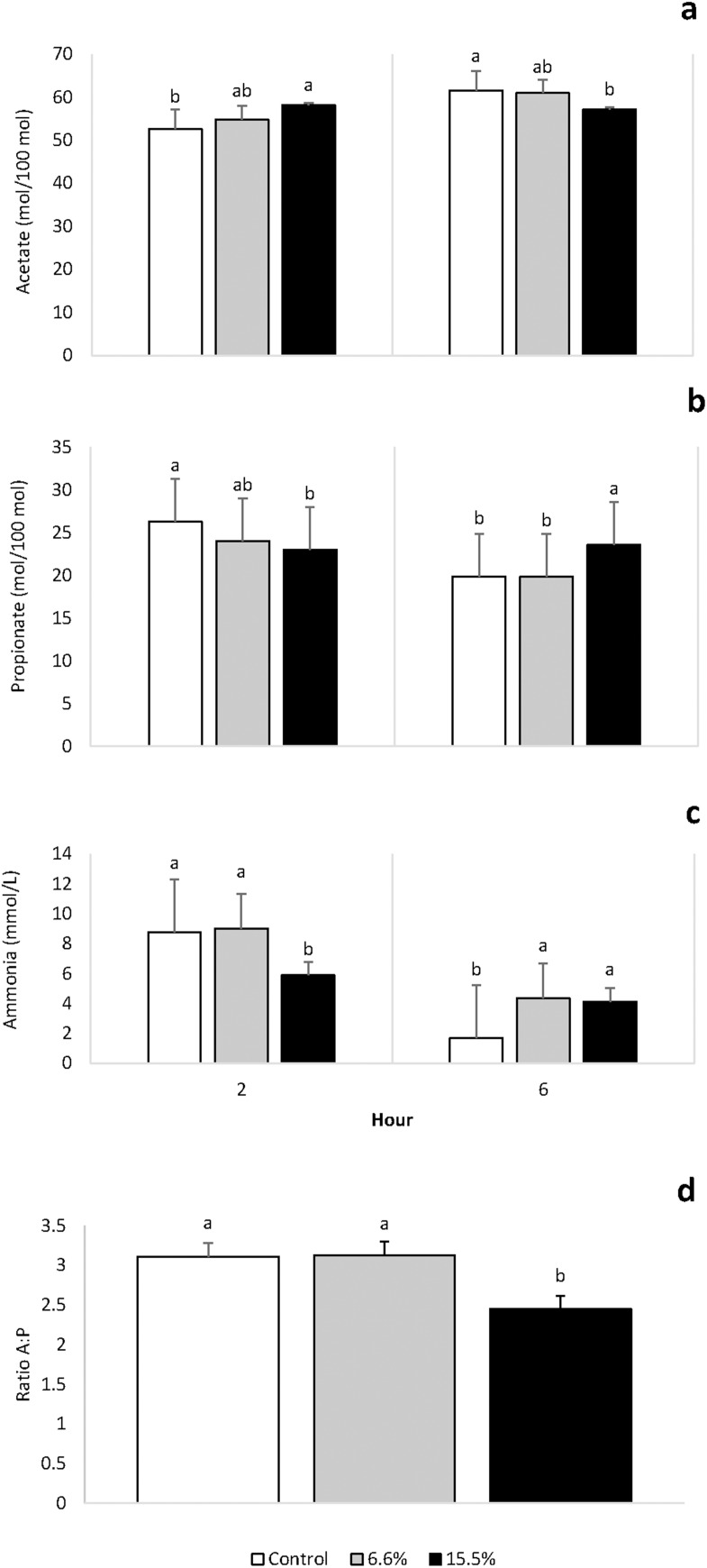
**Concentrations of VFAs present in ruminal fluid taken from dairy cows fed a forage mixed ration supplemented with *Tithonia diversifolia* which replaced other feed ingredients a:** Acetate at 2 h and 6 h after feeding, **b**: propionate 2 h and 6 h after feeding, **c**: acetate:propionate 6 h after feeding, **d**: ammonia 2 h and 6 h after feeding.

VFA concentrations are shown in Table 6. The inclusion of *T*. *diversifolia* had no effect (*P ≥* 0.094) on butyrate or valerate concentrations. There was a time effect (*P* = 0.02) of *T*. *diversifolia* on the total VFA concentration where the total VFA were greater at 2 h than 6 h. The concentration of BCVFA was greater (*P* < 0.001) with the inclusion of *T*. *diversifolia*. There was a treatment × time interaction for acetate, propionate, A:P and ammonia (Fig 2).

**Table 6:** Ruminal fermentation characteristics from dairy cows fed two concentrations of *T*. *diversifolia* in sugarcane-based diets.

	TREATMENTS^1^				*P-*value		
Item^2^	Control	6.5% T	15.4% T	SEM	Treatment	Time	Treatment × Time
Total VFA (mmol/L*)*	101.9	102.8	103.3	1.60	0.821	0.0214	0.1192
VFA, mol/100 mol							
Acetate (A)	57.1	57.9	57.7	1.07	0.859	0.0007	0.0091
Propionate (P)	23.1	21.9	23.3	0.72	0.375	0.0004	0.0071
Butyrate	15.2	14.5	13.2	0.61	0.094	0.1999	0.2904
Valerate	1.99	1.97	2.04	0.08	0.814	0.2823	0.9015
BCVFA^2^	2.66^b^	3.66^a^	3.79^a^	0.18	0.0002	0.1327	0.7531
A:P ratio	2.47	2.64	2.48	0.12	0.423	0.0003	0.0067
Ammonia (mmol/L)	5.24	6.68	5.01	0.37	0.008	<0.0001	0.0001

^a,b^Means within a row followed by different superscript letters differ significantly (*P* < 0.05).

^1^Control: 0% *Tithonia diversifolia*, 6.5%T: 6.5% *Tithonia diversifolia*, 15.4%T: 15.4% *Tithonia diversifolia*

^2^VFA = volatile fatty acids; BCVFA = Isobutyrate + Isovalerate.

**Fig 3:**
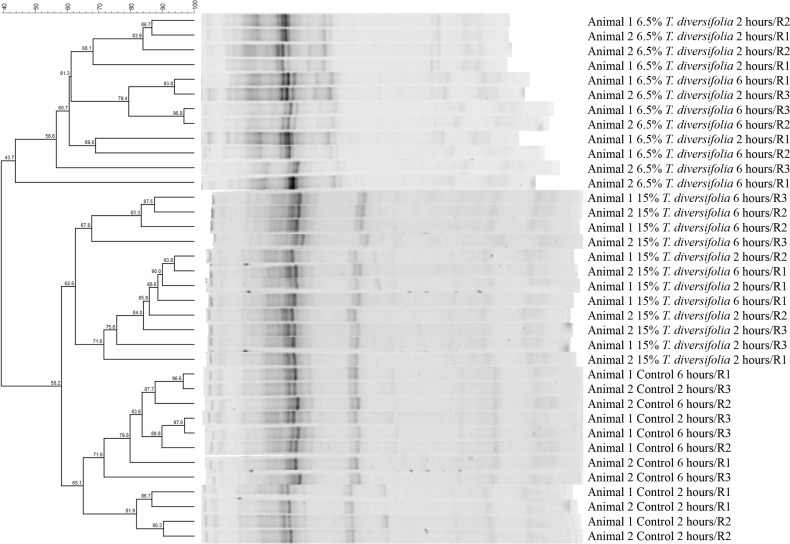
Electrophoretic profile of 16S rRNA gene sequence of rumen archaea from dairy cows fed sugarcane-based diets containing different concentrations of Tithonia diversifolia. The profiles were obtained after nested-PCR amplification of genomic DNA extracted from ruminal fluid. The UPGMA dendrogram was generated using BioNumerics 7.5.

**Fig 4:**
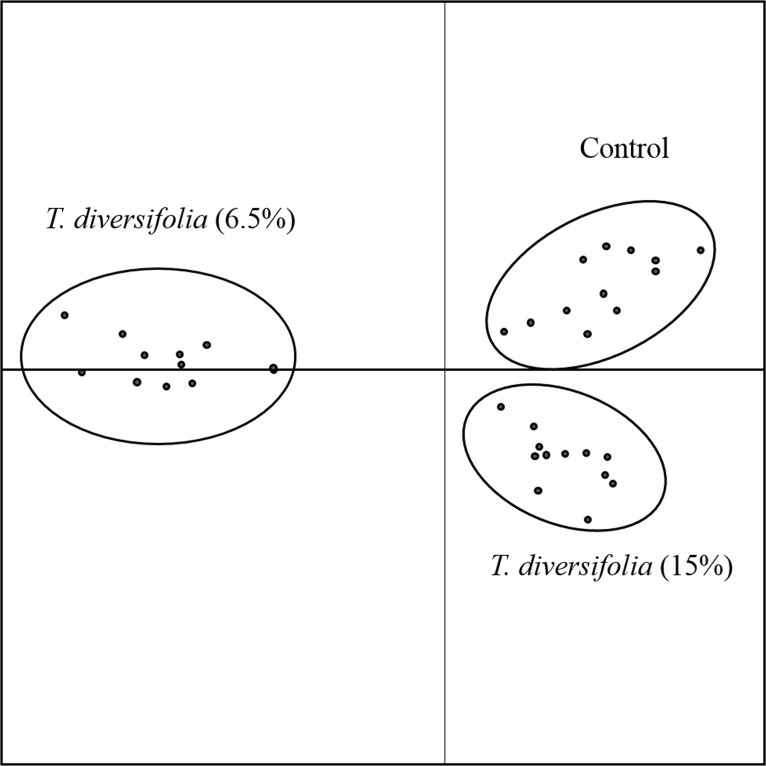
Principle co-ordinate plot of 16S rRNA gene sequence of rumen archaea from dairy cows fed sugarcane based diets containing different concentrations of *Tithonia diversifolia*. The plot was generated using BioNumerics 7.5.

There was no effect (P > 0.05) of the inclusion of *T*. *diversifolia* at any concentration on CH_4_ production (Table 7). The average mean production of CH_4_ was 385 g / day. The replacement of sugarcane and concentrates with *T*. *diversifolia* had no significant effect (*P ≥* 0.67) on CH_4_ production. However, there was a numerical increase in methane production as per g/day as the concentration of *T*. *diversifolia* increased which was not significant due to the large variation of data. Ruminal samples (liquid phase) analyzed by DGGE tended to separate the animals in three distinct clades grouped by the concentration of *T*. *diversifolia* included in each treatment (Fig 3). Amplification of the archaeal 16S rRNA V3 region revealed a range of 8–18 amplicons (average of 14.7 amplicons) that showed greater similarity between controls and the 15.4% *T*. *diversifolia* treatments, while the 6.5% *T*. *diversifolia* treatment had only 43.7% similarity with the other 2 treatments (Fig 3). Sampling time did not affect the microbial community composition within the same treatment. The difference in microbial community structure of dairy cows supplemented with different concentrations of *T*. *diversifolia* was also revealed by principle co-ordinate analysis (Fig 4).

**Table 7:** Methane production from dairy cows fed two concentrations of *T*. *diversifolia* in sugarcane-based diets.

	TREATMENTS^1^				
Item	Control	6.5% T	15.4% T	SEM	P value
CH_4_ (g/day)	365.7	380.8	409.8	61.1	0.88
CH_4_ (g/kg of DMI)	19.5	21.4	23.3	3.64	0.76
CH_4_ (g/kg of BW)	0.70	0.73	0.80	0.13	0.84
CH_4_ (g/kg of milk)	16.6	16.6	17.5	3.25	0.97

^1^Control: 0% *Tithonia diversifolia*, 6.5%T: 6.5% *Tithonia diversifolia*, 15.4%T: 15.4% *Tithonia diversifolia*

## Discussion

The tropical shrub, *T*. *diversifolia*, has been used across experiments as a high quality forage that can replace concentrates in ruminants [1–3,13]. Past research has shown that *T*. *diversifolia* presents as a suitable replacement for concentrates or as a supplement for nutritionally deficient diets with no effects or positive implications for production. However, no research has looked at the effects of *T*. *diversifolia* on in vivo CH_4_ production or archaeal population. As such, *T*. *diversifolia* was supplemented at up to 15.4% to determine its effects on production attributes and methane production.

Consistent with other literature the inclusion of *T*. *diversifolia* had no effect on DMI, milk production and milk composition [13]. *T*. *diversifolia* is high in energy and N with low cell wall contents [7,35]. These characteristics are known to increase DMI due to their great degradation rate in the rumen which leads to an increase in production [34]. However, DMI was not affected by the supplementation of *T*. *diversifolia* as the NDF content was similar across each diet.

There was no effect of dietary treatments on the concentrations of total SFA, MUFA and PUFA in milk fat. However, the concentrations of most trans-C18:1 isomers, including *trans*-11 C18:1 (vaccenic acid), were progressively increased in milk fat from cows fed *T*. *diversifolia*. The increase in milk fat *cis*-9, *trans*-11 CLA (rumenic acid) content observed in response to dietary inclusion of *T*. *diversifolia* is consistent with the increase in vaccenic acid, since most of this CLA isomer is produced endogenously in the mammary gland via the action of stearoyl-CoA (SCD) enzyme on vaccenic acid escaping from the rumen [36]. The accumulation of *trans*-C18:1 and non-conjugated C18:2 *isomers* in milk fat from cows fed *T*. *diversifolia* may indicate either an inhibition of rumen biohydrogenation of dietary PUFA by secondary compounds present in the forage or an increased supply of C18:2 n-6 and C18:3 n-3 from the diet [37]. Either way, the milk fat levels of biohydrogenation intermediates possessing anti-lipogenic activity (e.g. *trans*-10, *cis*-12 CLA and *trans*-9, *cis*-11 CLA) found in *T*. *diversifolia*-fed cows were insufficient to induce milk fat depression (Table 2), which was probably due to the very low lipid levels of the experimental diets (Table 1).

The type of biohydrogenation intermediate formed in the rumen and secreted in milk varies according to several dietary factors such as forage type and forage:concentrate ratio in the diet [32,38]. In particular, milk fat concentration of beneficial fatty acids such as rumenic, α-linolenic, and vaccenic acid have been shown to increase when pasture allowance is increased, and when conserved forages (notably hay) are replaced with fresh forage in the diet [39].

The inclusion of *T*. *diversifolia* had no effect on the total VFA concentrations. However, with increasing proportions of *T*. *diversifolia* replacing up to 20.8% of sugarcane and up to 11.4% of concentrates, there was a resultant increase in the A:P at 6 hours after feeding. When diets great in structural carbohydrates (cellulose, hemicellulose) are fed to ruminants, ruminal fermentation alters to favor the production of acetate and decreases the production of propionate. The increase in acetate production resulting from the inclusion of *T*. *diversifolia* in the diet can then result in an increase in CH_4_ production [40, 41]. The lack of statistical significance of methane production may be attributed to the poor sensitivity of this measurement technique. In the current experiment the rate of SF_6_ released from the tracer was 2.63 ± 0.94 mg/d, which according to Moate et al. [42] a minimum release rate of 3.0 mg/day is recommended. This variation in SF_6_ release rate may also have resulted in the large variation of data as Deighton et al. [43] recommends a uniform set of permutation tubes be used.

In a recent experiment, Terry et al. [14] demonstrated that in vitro VFA concentration increased when *T*. *diversifolia* was supplemented at 15.2% DM replacing fresh sugarcane and concentrates. These last authors also reported an increase in the A:P and CH_4_ production with increasing concentrations of *T*. *diversifolia*. In the in vivo experiment there was no effect (*P* = 0.82) of the inclusion of *T*. *diversifolia* on total VFA and as such, no increase in production paramaters. Delago et al. [7] found that *T*. *diversifolia* possessed methane reducing properties when supplemented at 30% into a star grass (*Cynodon nlemfuensis*) based diet. Authors indicated that this was due to the presence of secondary metabolites in *T*. *diversifolia* such as condensed tannins and saponins. In the current study, the presence of antinutrients was not evaluated which presents as a difficulty when assessing their effects on CH_4_ production.

A novel finding of this research was that PCR-DGGE profiling showed clear differences between archaeal communities according with the amounts of *T*. *diversifolia* included in the diet. Despite the genetic diversity of the archaeal populations from each treatment there was no differences in species richness and no effect on CH_4_ production among treatments. These results reinforce the idea that changes in diversity of a community cannot always be linked with changes in activity (and vice versa) due to the functional redundancy and relative abundances of the microorganisms involved. Enteric methane production can be affected by a direct effect of dietary constituents or feed additives on the methanogenic population concentrations or by shifts in the bacterial community that alter the availability of substrates to the methanogens (e.g. formate and hydrogen)[44]. Further work will be needed to identify the major microbial populations affected in the treatments containing *T*. *diversifolia* in the diet.

A new study has found that dominant archaeal groups (*Methanobrevibacter gottschalkii*, *Methanobrevibacter ruminantium*, *Methanosphaera* spp. and two *Methanomassiliicoccaceae* affiliated groups) accounted for 89.2% of all archaeal communities present globally in ruminants and camelids [45]. It also found that the bacterial community structures were mostly determined by diet and host with little changes observed within archaeal and protozoal populations. Interestingly, the electrophoretic profile of 16S rRNA gene sequence of the rumen archaea showed that the rumen populations of cows fed the control diet and the 15.4% *T*. *diversifolia* diet were more similar than the 6.5% *T*. *diversifolia* diet. This unusual finding suggests that dose rate of *T*. *diversifolia* has a non-linear effect on ruminal micro-organisms. To further improve the study deep sequencing of the archaeal community or quantification of abundant archaeal groups could help characterize more precisely the influence of the diet on methanogens. This could aid in identifying the link between the different diets and their corresponding archaeal population.

## Conclusion

The replacement of up to 20.8% of sugarcane and up to 11.4% of concentrates (DM basis) with *T*. *diversifolia* had no effect on DMI, milk production or composition, N balance, or CH_4_ production. There was also no increase of total VFA concentration as hypothesised from previous experiments. Three distinct methanogenic populations were characterized for each treatment. From this experiment it can be concluded that *T*. *diversifolia* can be used as a suitable feed ingredient by replacing forage and concentrates without negative effects on performance and methane production in dairy cows under these experimental conditions.
